# pIChemiSt — Free Tool for the Calculation of Isoelectric Points of Modified Peptides

**DOI:** 10.1021/acs.jcim.2c01261

**Published:** 2022-12-27

**Authors:** Andrey I. Frolov, Sunay V. Chankeshwara, Zeyed Abdulkarim, Gian Marco Ghiandoni

**Affiliations:** † Medicinal Chemistry, Research and Early Development, Cardiovascular, Renal and Metabolism (CVRM), BioPharmaceuticals R&D, AstraZeneca, Gothenburg, Sweden; ‡ Early Chemical Development, Pharmaceutical Sciences, BioPharmaceuticals R&D, AstraZeneca, Gothenburg, Sweden; § Augmented DMTA Engineering, R&D IT, AstraZeneca, Cambridge, U.K.

## Abstract

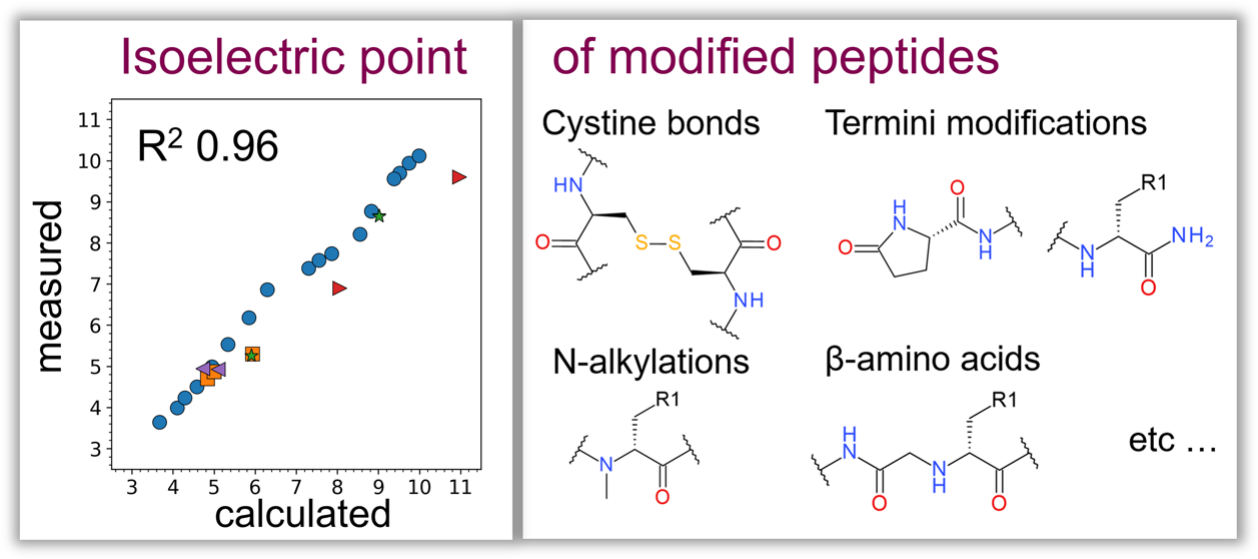

The isoelectric point (pI) is a fundamental physicochemical property of peptides and proteins. It is widely used to steer design away from low solubility and aggregation and guide peptide separation and purification. Experimental measurements of pI can be replaced by calculations knowing the ionizable groups of peptides and their corresponding p*K*_a_ values. Different p*K*_a_ sets are published in the literature for natural amino acids, however, they are insufficient to describe synthetically modified peptides, complex peptides of natural origin, and peptides conjugated with structures of other modalities. Noncanonical modifications (nCAAs) are ignored in the conventional sequence-based pI calculations, therefore producing large errors in their pI predictions. In this work, we describe a pI calculation method that uses the chemical structure as an input, automatically identifies ionizable groups of nCAAs and other fragments, and performs p*K*_a_ predictions for them. The method is validated on a curated set of experimental measures on 29 modified and 119093 natural peptides, providing an improvement of *R*^2^ from 0.74 to 0.95 and 0.96 against the conventional sequence-based approach for modified peptides for the two studied p*K*_a_ prediction tools, ACDlabs and pKaMatcher, correspondingly. The method is available in the form of an open source Python library at https://github.com/AstraZeneca/peptide-tools, which can be integrated into other proprietary and free software packages. We anticipate that the pI calculation tool may facilitate optimization and purification activities across various application domains of peptides, including the development of biopharmaceuticals.

## Introduction

### Peptides as Therapeutics

The number of FDA-approved peptide therapeutics (PTs) has been steadily growing over the past decade.^1^ Peptides and peptidomimetics fill an intermediate niche between conventional small molecules and large biologic therapeutics.^2^ Compared to small molecules, PTs allow targeting of shallow pockets and are usually less promiscuous. Compared to biologics, they are less immunogenic and have lower production costs.

### Chemically Modified Peptides Are Widely Spread in Nature and Industry

Bioactive peptides originate from various natural sources (e.g., endogenous hormones,^3^ toxins,^4^ etc.), hit-finding approaches (e.g., phage display),^5,6^ and rational design (e.g., epitope mimetics).^7,8^ Peptide starting points are often modified with nCAAs^9^ to modulate the interaction with the target surface^10^ and improve *in vivo* stability and oral bioavailability.^11−13^ Short cross-linked peptides mimicking interaction epitopes are often designed for the inhibition of protein–protein interactions (PPIs).^7,8,10,14,15^ Peptides chemically linked with a cargo molecule can serve as targeted delivery vehicles to the tissues of interest.^16^ In addition to these applications, medicinal chemists are also constantly searching for noncanonical peptide mimetics and peptide-hybrids, such as peptoids,^17^ peptidomimetics,^18^ foldamers,^19^ and peptide-natural product combinations for their use as therapeutics.^20^ In turn, living organisms provide an immense source of toxins, venoms, and other bioactive peptides that are often highly modified.^4^ Additionally, nature provides the whole machinery of chemical modification of canonical amino acids with various post-translational modifications (PTMs).^21,22^

### Challenges with Peptide Purification, Separation, and Immunogenicity

Handling peptides with natural and noncanonical amino acids in the lab faces practical challenges: their synthesis often requires multiple steps resulting in impurities that are structurally very similar,^23^ making purification difficult and time-consuming.^24,25^ Reverse-phase HPLC is widely employed in peptide purification but is burdened by its own challenges related to strong retention of hydrophobic peptides, sustainability, and cost, particularly on a large scale.^26^ Conversely, ion-exchange (IEX) chromatography can be used to purify peptides with similar lipophilicity but differing in ionization states using appropriate mobile phase conditions.^27−31^ Note that peptide ionization has a significant effect on solubility and aggregation at different pH^32^ that may result in undesired immunogenicity and toxicity.^33−36^

### Isoelectric Point to Address the Challenges

Issues related to the physical properties of peptides can often be addressed by analyzing their charge states. First, keeping the pH of the mobile phase away from the isoelectric point, for example, by adding acidic ion-pairing agents,^37^ reduces undesired lipophilic interaction with the column during purification.^24,32^ Second, in some cases, impurities, even being structurally similar to the desired product, differ by their isoelectric point or, more generally, by their net molecular charge at different pH. By analyzing synthesis and mass spectrometric data, it is possible to make a qualified guess as to which amino acid could have failed to couple or coupled unprompted, that is, missing/added in the sequence.^38−40^ A wise selection of the pH of the mobile phase, based on the difference in calculated pI of the target peptide and the anticipated byproducts, allows the separation of chromatographic peaks even for structurally similar compounds.^24,31^ Overall, one can improve peptide solubility and physical stability of peptide solutions by designing the isoelectric point away from the relevant pH range.^32^ In addition, predicting ionization states are often attempted to rationalize peculiar observations in biological and biophysical assays. For instance, Zapadka et al. associated a highly unusual pH-induced switch in GLP-1 aggregation kinetics to the protonation/deprotonation of the N-terminus.^33^

### Lack of Tools Predicting Isoelectric Points of Modified Peptides

Despite the evident need for pI prediction methods for modified peptides, there is still a lack of free and robust tools. There are tools that provide isoelectric point predictions for natural peptides in the form of web services,^41−43^ free or licensed standalone software.^44−47^ A few more tools support some common PTMs but only in a limited number.^48,49^ In addition, Bjerrum et al. published a tool that can handle noncanonical amino acids.^46^ Its limitations, however, are that the method relies on licensed software^50^ for structure-to-sequence conversion and only implements a small set of 17 rules to predict p*K*_a_ of noncanonical side chains. Moreover, the lack of a front-end service to facilitate its usage further hinders its uptake.

### pIChemiSt—New Method Predicting Isoelectric Points of Modified Peptides

In the present work, we describe the development of an open source tool called pIChemiSt for isoelectric point calculation of natural and modified peptides. The tool integrates two programs for the prediction of ionization constants of nCAAs, namely, the licensed ACDlabs^51^ and pKaMatcher developed in-house to avoid dependencies on proprietary software. The latter is validated to cover a set of around 300 noncanonical amino acids available for peptide synthesis in the AstraZeneca chemistry stock. We also describe the curation of a data set of experimental isoelectric point values for modified peptides from the Reaxys database.^52^ Finally, we demonstrate that the predictions from our tool provide substantially superior accuracy compared to the plain sequence-based approach. The tool is released free of charge on GitHub.^53^ In addition, an associated web server is under development and will be described in a future publication.^54^

## Methods

### Calculating Charge versus pH Curves

**Figure 1:**
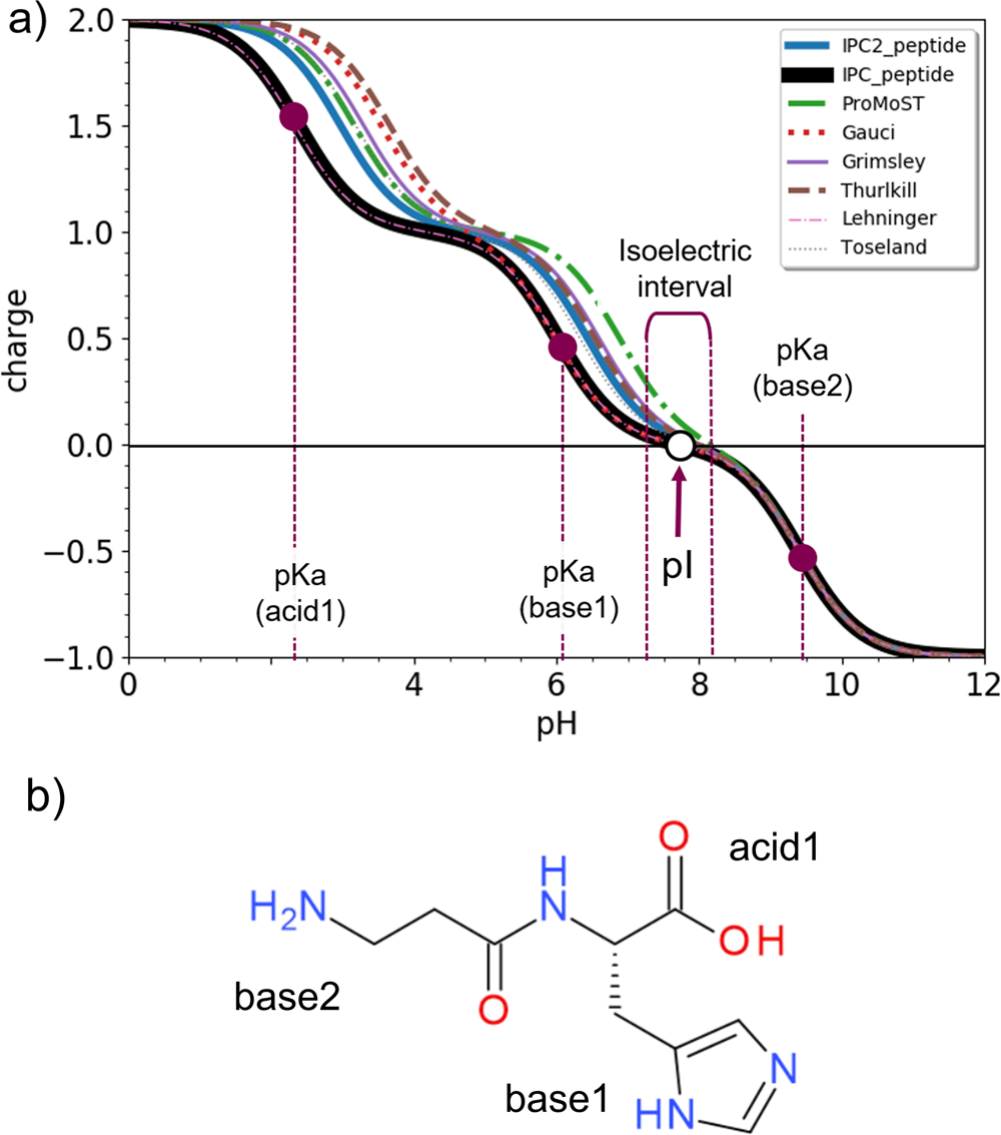
(a) Charge versus pH curves resulting from the Henderson–Hasselbalch equations for the dipeptide shown in (b). Different curves correspond to various predefined p*K*_a_ sets of canonical amino acids. The magenta dots (p*K*_a_ (acid1), p*K*_a_ (base1), p*K*_a_ (base2)) mark the inflection points, which correspond approximately to the p*K*_a_ values of the ionizable groups for the case of the IPC_peptide p*K*_a_ set. The white dot corresponds to the isoelectric point of the molecule. The isoelectric interval denotes the span of pH within which the predicted peptide charge is negligible. This parameter may be used instead of the calculated pI when the latter is poorly defined. The variation between different p*K*_a_ sets allows judging the uncertainty of the calculated pI, which is reported as mean value ± standard deviation in the output. The group identified as ‘base2’ belongs to an N-terminus of a noncanonical amino acid; therefore, its p*K*_a_ does not belong to the predefined p*K*_a_ sets and is calculated on the fly. (b) Chemical structure of a peptide with three ionizable centers, C-terminal acid, basic N-terminal amine, and the histidine side chain.

The linear combination of the Henderson–Hasselbalch equations is commonly used for the calculation of protein or peptide charges as a function of pH (eq 1). The equation requires a set of apparent p*K*_a_ values and types of dissociation (acid or base) of all ionizable groups in the molecule
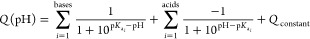
1where the two sums run over all basic and acidic groups of the molecule, correspondingly; p*K*_a_*i*__ is negated logged acid-ionization constant of the acid or the conjugated acid of the base; *Q*_constant_ represents the permanent charge of the molecular entity associated with the constantly ionized molecular groups unless the molecule undergoes chemical decomposition, such as quaternary ammonium and alkyl pyridinium cations. The charge versus pH curves are illustrated in Figure 1.

### Calculation of Isoelectric Point

The isoelectric point, by definition, is the pH at which the net molecular charge is zero. Therefore, pI can be found by solving eq 2 numerically, where *Q*(pH) represents the net molecular charge
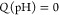
2

In this work, we use the bisection method with an initial pH interval that goes from 0 to 14 and a charge tolerance of 0.01e. There are examples where the *Q*(pH) curve does not intersect the zero line, for example, when there are no ionizable centers in the molecule or there are either only basic or acidic residues in the sequence. In these cases, pI is technically not defined, and hence, no value is returned. We also capture the cases when the curve approaches the zero line from one side asymptotically. In these cases, we set a threshold of 0.05e as an indicator that the molecule “becomes uncharged”. Another case where pI is poorly defined is when there is a large separation between p*K*_a_ of basic and acidic groups in the peptide. In this case, the *Q*(pH) curve crosses the zero line, but the slope of the curve is negligible, making it challenging to pick a pI value from a large pH interval. To characterize such cases, we introduce the concept of “isoelectric interval”, which is defined as a pH span where the net charge of the molecule can be considered negligible. Hence, we set the following interval for the charge being between −0.05 and 0.05e.

### Determination of Ionizable Groups in the Molecule

**Figure 2:**
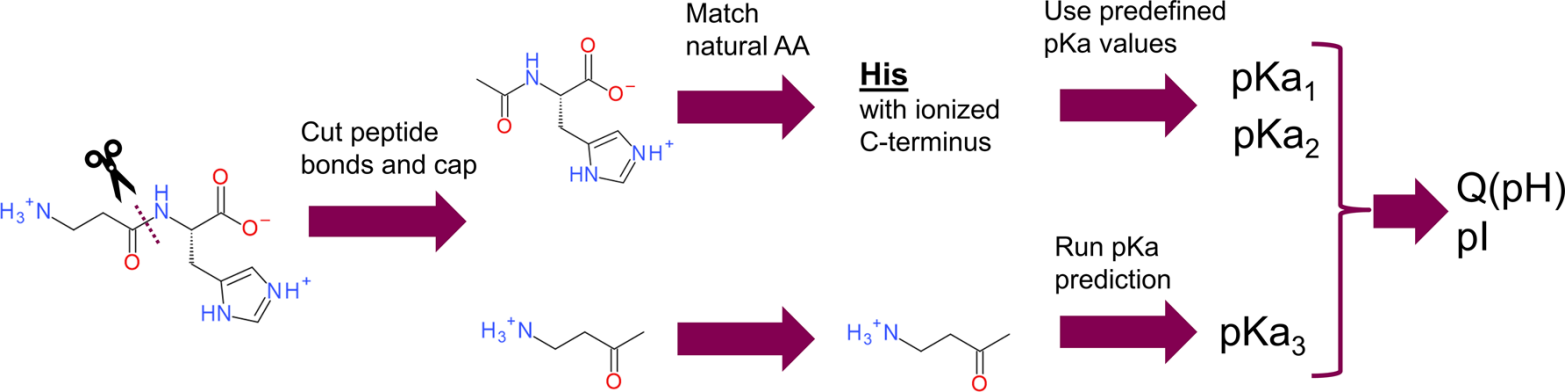
Workflow of the pI calculation. The input chemical structures are analyzed, and all secondary and tertiary amide bonds are cleaved. The resulting amines and aldehydes are capped with acetyl and methyl groups, respectively. The resulting fragments are matched with the templates of canonical amino acids. The corresponding p*K*_a_ values of the side chain and N- or C- termini are attributed from a look-up table. When a fragment is not recognized as a canonical amino acid, the method predicts ionizable centers and their corresponding p*K*_a_ values. The p*K*_a_ values of noncanonical fragments and canonical amino acids are combined and inserted into eqs 1 and 2 to obtain the charge versus pH curve, isoelectric point, and interval.

The input structure is processed as described in Figure 2. First, we cut all peptide bonds and cap the resulting N-termini with acetyls and C-termini with methyl ketone groups. The capping allows modeling the effect of peptide backbone on predicted p*K*_a_ values of side chains, which might be significant as the carbonyl groups have strong electron-withdrawing character. Second, we match the canonical amino-acid side chains using a built-in set of SMARTS patterns (see Table S1 in SI). Note that the SMARTS patterns list hydrogens, tautomeric and ionization forms of each natural amino acid explicitly. Ionizable N-terminus amines and conventional C-termini (carboxylic acid and primary amide) are also encoded in the SMARTS patterns. The corresponding p*K*_a_ values of ionizable side chains and termini are taken from the predefined sets (see the following section). Third, for the fragments that have no matches against the SMARTS patterns, we run automated p*K*_a_ predictions (see the following sections). Finally, we merge the p*K*_a_ sets from natural amino acids and unmatched fragments and use them to calculate charge versus pH curves and pI. Note that individual amino acids, where both termini are ionized, are not encoded in the SMARTS patterns, and thus, they are treated as noncanonical fragments by the software.

### p*K*_a_ Sets of Natural Amino Acids

There are many p*K*_a_ data sets reported for natural amino acids, either derived from experimental measurements (Grimsley,^55^ Thurlkill,^56^ Toseland^57^), trained to reproduce experimental data (IPC_Peptide, IPC2_Peptide, IPC_Protein, and IPC2_Protein^42,43^), or obtained by combining experimental data with Hammett–Taft-derived electronic effects (initial Bjellqvist,^41,58^ extended Bjellqvist,^59^ Gauci^60^). The nature of other sets commonly used as benchmarks could not be deduced from the literature (ProMoST,^48^ DTASelect,^61^ Rodwell,^62^ EMBOSS,^63^ Nozaki^64^). The p*K*_a_ values from the textbooks most likely refer to the experimental data of individual amino acids derived by potentiometric titration (Lehninger,^65^ Solomons^66^). Most p*K*_a_ sets contain nine p*K*_a_ values, two for N- and C-termini and seven for ionizable amino-acid side chains. There are also sets where the position of amino acids within a sequence influences their p*K*_a_ values: Bjellqvist et al. introduced a 17-parameter set where the p*K*_a_ values of the N- and C-termini vary depending on the type of the termini side chains.^59^ Later, Gauci et al.^60^ introduced two empirical values to the extended Bjellqvist set^59^ as they observed a systematic deviation for predictions when the Cysteine or Asparagine were at the N-terminus. Note that the p*K*_a_ values in the Gauci set are not provided in their original publication; however, they are included in the source code of the pIR software.^44,45^ Finally, ProMoST^48^ uses a more advanced algorithm where the p*K*_a_ values of side chains vary between C- and N-termini and the rest of the sequence results in around 56 p*K*_a_ values. The origin of the p*K*_a_ values implemented in ProMoST is not disclosed in its publication.^48^ The sets implemented in this work are listed in Table 1.

**Table 1:** p*K*_a_ Sets Used in the Study

	N-term.	C-term.	C	Y	D	E	H	K	R	origin
IPC_peptide	9.564	2.383	8.297	10.071	3.887	4.317	6.018	10.517	12.503	machine learning
IPC2_peptide^43^	7.947	2.977	9.439	9.153	3.969	4.507	6.439	8.165	11.493	machine learning
Gauci^44,60^^a^	6.50–8.36	3.55–4.75	9.0	10.0	4.05	4.45	5.98	10.0	12.0	isoelectric focusing, Taft calculations, literature data
Grimsley^55^	7.7	3.3	6.8	10.3	3.5	4.2	6.6	10.5	12.04	experimental data (primarily NMR) of folded proteins
Toseland^57^	8.71	3.19	6.87	9.61	3.6	4.29	6.33	10.45	12.0	experimental p*K*_a_ data (primarily NMR) of folded proteins
Thurlkill^56^	8.0	3.67	8.55	9.84	3.67	4.25	6.54	10.4	12.0	potentiometric titration of pentapeptides
Lehninger^65^^b^	9.69	2.34	8.33	10.0	3.86	4.25	6.0	10.5	12.4	potentiometric titration of amino acids
ProMoST^48^^c^	6.67–8.36	3.17–3.98	8.00–9.00	9.34–10.34	3.57–4.57	4.15–4.75	4.89–6.89	9.8–10.30	11.5–12.5	not disclosed

a The Gauci set is based on the extended Bjellqvist set^59^ with two additional p*K*_a_ values for N-terminal Cys and Asn. The p*K*_a_ values in the extended Bjellqvist set were obtained from the experimental measures of the pI of the human carbonic anhydrase enzyme. Ionization constants of C-terminus, Asp and Glu side chains, were calculated using the Taft equation. The p*K*_a_ of the N-terminus was derived from literature. No data were reported for the remaining amino acids as their p*K*_a_ values fell outside the pH ranges considered in the study.^58^ However, these were listed in the subsequent publication, where they were derived from other literature sources.^59^ The p*K*_a_ of the N- and C- termini in this set depends on the type of amino acid.

b The p*K*_a_ values of the side chain and N- and C-termini are reported for each amino acid. The origin of the data is unclear. However, it may result from potentiometric titration. The values for the N-terminus and C-terminus are averaged between all amino acids.

c The p*K*_a_ of the N- and C- termini depends on the type of amino acid. The p*K*_a_ values of the side chains depend on the location of amino acids, where values at N- and C-termini differ from the rest of the sequence. We provide the span of values in the table. The p*K*_a_ values can be found in publicly available data sources on the web.^53,67,68^

### Prediction of p*K*_a_ for Noncanonical Amino Acids

When a fragment is not recognized as a canonical amino acid, we analyze whether it has ionizable or constantly charged groups and predict the p*K*_a_ of each ionizable center. Here, we implemented two options for such a purpose: the licensed ACDlabs^51^ GALAS method and an in-house open source tool called pKaMatcher, which can be used as an alternative to ACDlabs. Sometimes predictions include p*K*_a_ of groups that change their ionization only at extreme pH ranges, which fall outside those typically evaluated experimentally (pH 2–12).^69,70^ We deliberately excluded these extreme p*K*_a_ values from calculations as follows: p*K*_a_ of acids should be below 12 and p*K*_a_ of bases should be above 2 to be included in the calculation. Additionally, we set the lower limit for the acidic p*K*_a_ to be −5 and the upper limit for the basic p*K*_a_ to be 15. These limits are meant to keep strong relevant acids and bases in the calculation, for example, sulfate or guanidinium groups.

### Prediction of p*K*_a_ with ACDlabs

The tool executes the Percepta Batch (perceptabat) module of ACDlabs.^51^ We observed a misassignment of the type of p*K*_a_ equilibria (acid versus base) for one of the non-natural amino acids while testing the CLASSIC algorithm, which is based on the Hammett–Taft equations. Therefore, we adopted the GALAS algorithm, despite being less documented. From the perceptabat output, we read all the predicted p*K*_a_ values and the corresponding type of equilibria (acid or base) needed in eq 1.

### Prediction of p*K*_a_ with pKaMatcher

We developed a simple SMARTS pattern-matching approach for assigning p*K*_a_ values of noncanonical amino acids and unknown fragments. Currently, there are 56 SMARTS patterns in the list covering the most used ionizable groups in medicinal chemistry. The algorithm attempts to match every SMARTS pattern on every unknown amino acid. In the case of a match, the corresponding p*K*_a_ value and the type of equilibrium are added to the list of p*K*_a_ utilized in eq 1. For each SMARTS pattern, the algorithm keeps track of which atom of the matched substructure is ionizable, i.e., binds or releases a proton. The p*K*_a_ value is excluded if any previous SMARTS pattern already matched the ionizable atom. Some SMARTS patterns match functional groups with two ionizable centers, for example, a phosphate ion. In such cases, two p*K*_a_ values and the corresponding types of equilibria are added to eq 1. An initial list of SMARTS patterns and the corresponding p*K*_a_ values were adopted from Dimorphite-DL,^71^ where they were derived from experimental data. Later, we adjusted the definitions of most of the SMARTS to improve their use for our particular purpose by removing definitions that were too generic (e.g., aromatic_nitrogen_unprotonated/protonated, primary/secondary/tertiary amines), by adding more functional groups of medicinal chemistry relevance (e.g., tetrazole, imidazole, substituted phenols), or by excluding patterns with p*K*_a_ outside the relevant pH range (e.g., alcohols with acidic p*K*_a_ around 14). For the newly added SMARTS patterns, we set the p*K*_a_ values predicted by ACDlabs. The described SMARTS patterns (see Table S1 of SI) were sufficient to cover the ionizable groups present in 93 non-natural amino acids ready for solid-state peptide synthesis available from the AstraZeneca chemistry inventory.

### Constantly Ionized Residues

Some molecular groups have a constantly ionized charge that would not get a predicted p*K*_a_ value. However, these groups would still affect the net charge of the molecule and the predicted pI as a consequence. In the current implementation, the four-valent nitrogens are identified as constantly ionized.

### Sequence-Based pI Calculations

These predictions were performed using a FASTA sequence-based script that is included in the pIChemiSt repository. The sequence-based algorithm is identical to the one of pIChemiSt with the only exception that the canonical amino acids are derived from the single letter FASTA sequences. Both N- and C-termini are assumed to be ionizable. The contribution from the side chains of noncanonical amino acids denoted as *X* in the sequence is ignored in the calculations. Predictions are reported as mean pI values averaged over the data obtained with the same predefined sets of p*K*_a_ values of canonical amino acids as in the case of pIChemiSt calculations.

### Experimental Data Sets for Validation

**Figure 3:**
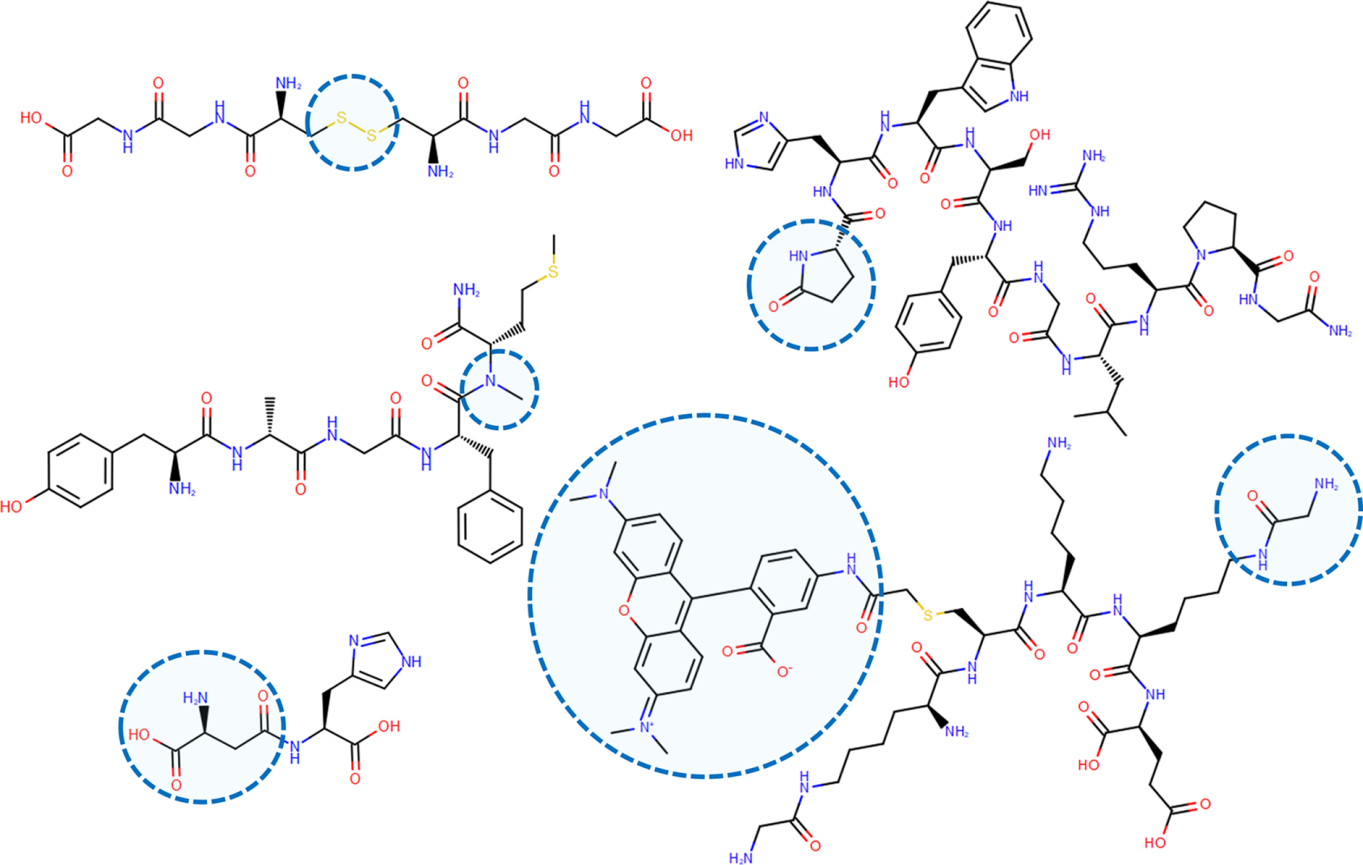
Representative compounds from the validation set with measured pI data. Blue circles highlight noncanonical modifications: cystine bond,^73^ β amino acid,^72^ N-methylation,^76^ pyroglutamic acid,^75^ derivative of the tetramethylrhodamine 5-iodoacetamide dye and glycyl-modified lysine side chain.^74^

Bjerrum et al.^46^ validated their method on about 100 pI data points of peptides with noncanonical modifications that were retrieved from the Reaxys database.^52^ We used the Reaxys IDs provided by Bjerrum et al.^46^ and retrieved the data from Reaxys for our validation. However, while browsing the original references, we discovered that many data points that are attributed as experimental data in the Reaxys database were not measured but calculated. We carefully inspected the sources in Reaxys and discovered that only five publications contained experimental data,^72−76^ from which we constructed a curated data set. We used the curated data set for validating our software for the case of modified peptides. We also evaluated the tool against the IPC2_peptide assembled set of experimental data of natural peptides as an additional validation.^43^ The sequences were converted to two-dimensional (2D) structures with ionized N- and C-termini. Structures from the validation set are exemplified in Figure 3.

### Dependencies

pIChemiSt is written in Python3, utilizes RDKit^77^ for cheminformatics processing and Matplotlib^78^ for graph plotting. The auxiliary scripts of the distribution utilize BioPython^79^ functions to read in files in “.fasta” format.

## Results

**Figure 4:**
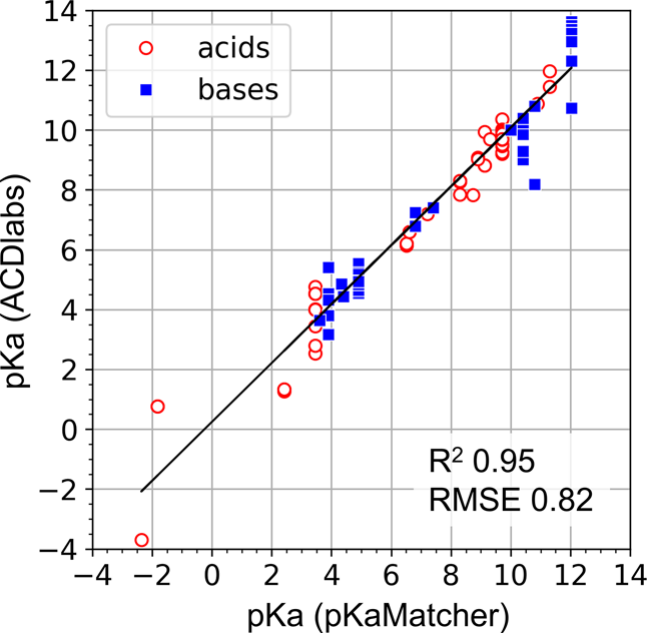
Validation of p*K*_a_ predictions by pKaMatcher against ACDlabs using a set of 93 ionizable noncanonical amino acids available in the AstraZeneca stock. The coefficient of determination and root-mean-squared deviation are reported at the top of the plot.

We validated our in-house pKaMatcher against the ACDlabs tool using a set of 271 noncanonical amino acids available for solid-phase synthesis in the AstraZeneca stock. Only 93 amino acids were identified as ionizable by both tools. The correlation between predicted p*K*_a_ values is shown in Figure 4.

**Figure 5:**
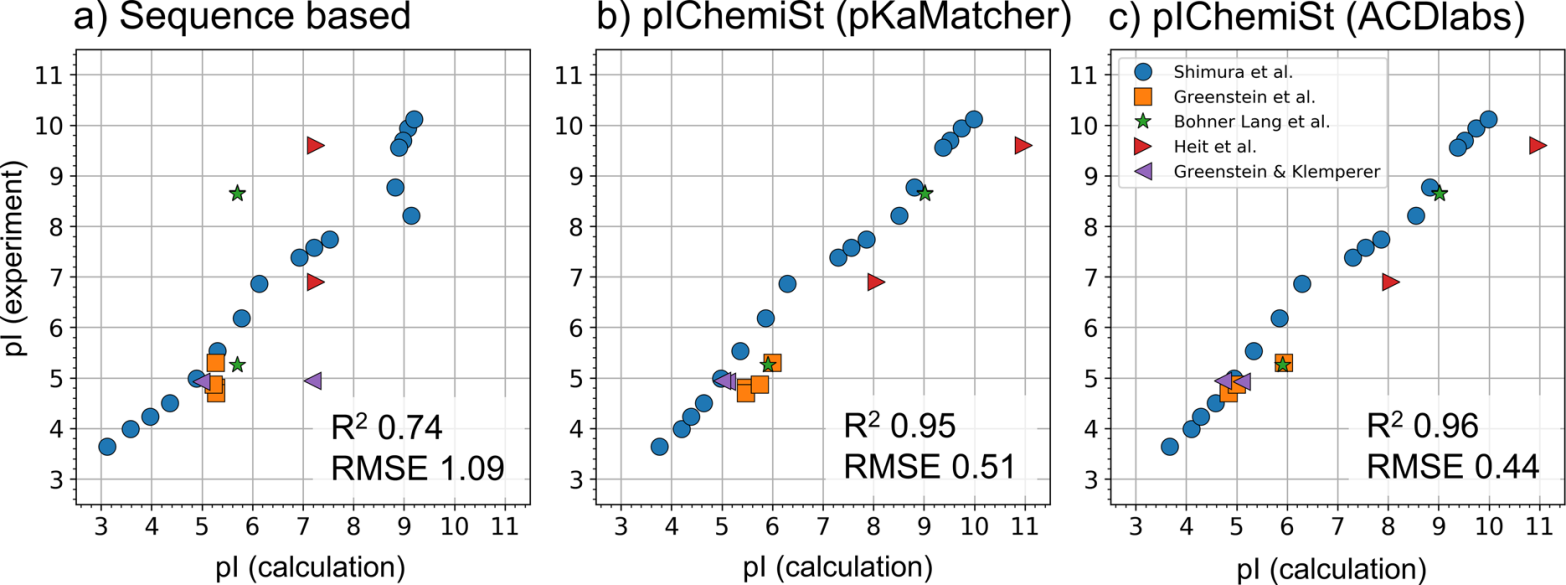
Validation of pIChemiSt on a set of modified peptides. (a) Predictions by the sequence-based model where all noncanonical amino acids and N- and C-termini modifications are ignored; (b) Predictions by pIChemiSt using pKaMatcher as a tool for calculating p*K*_a_ of noncanonical amino acids; (c) Same as b but with ACDlabs for p*K*_a_ calculations. The coefficient of determination and root-mean-squared deviation are denoted on the plots. Experimental data are from the following refs (72−76).

**Figure 6:**
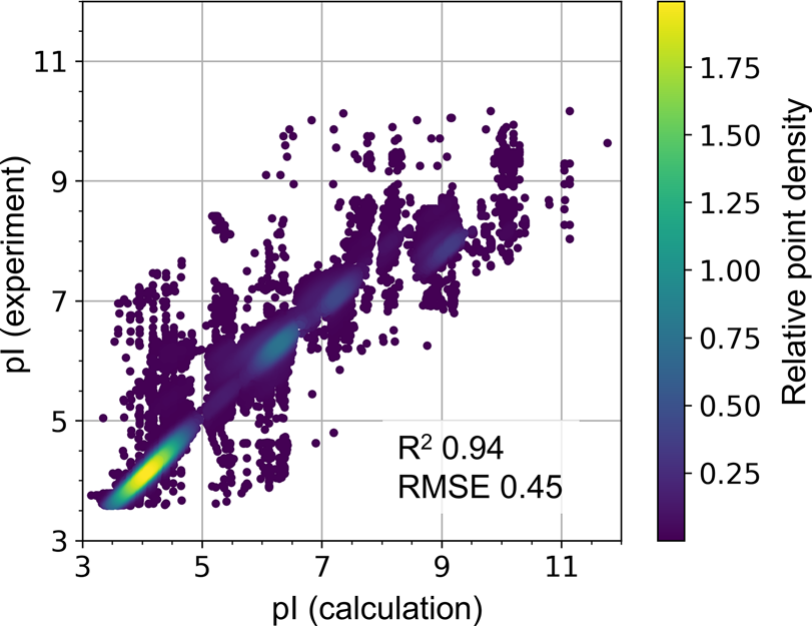
Validation of pIChemiSt on IPC2_peptide set of 119093 natural peptides. The coloring is proportional to the density of the points on the plot with lighter color representing denser regions.

We validated pIChemiSt on a curated data set of isoelectric points of modified peptides. The results are shown in Figure 5. In addition, we have validated pIChemiSt on a set of isoelectric points of natural peptides from the IPC2_peptide data set (see Figure 6).^43^ The sequences from IPC2_peptide set were first converted to chemical structures.

## Discussion

### Importance of Predicting p*K*_a_ of Noncanonical Amino Acids

The accuracy of pI calculations significantly improves when we introduce automated p*K*_a_ predictions for noncanonical amino acids and termini modifications. Moving from the plain sequence-based to the structure-based predictions, *R*^2^ improved from 0.74 to 0.96 and 0.95 with RMSE shift from 1.09 to 0.44 and 0.51, using ACDlabs and pKaMatcher for p*K*_a_ predictions, correspondingly. One of the major limitations of sequence-based predictions is that they fail to capture substantial differences between neighboring analogues. For instance, the luteinizing hormone-releasing hormone (LHRH), which has a primary amide at the C-terminus, and its close analogue with a carboxylic acid at the C-terminus, are predicted to have identical pI by the sequence-based approach. However, their pI differs by 2.7 according to the experimental measurements by Heit et al.^75^ The problem is that the plain FASTA sequence does not allow for decoding the differences in termini of the two peptides. In contrast, the chemical structure-based approach captures the difference and produces an estimation that is close to the experimental value (2.9 as calculated with both ACDlabs and pKaMatcher p*K*_a_ predictors). Another example regards a case when two dipeptides, α- and β- aspartyl-histidine, appear to have almost identical pI values experimentally. However, their pI values predicted by the sequence-based approach differ by 2.2 units. In particular, the automatically generated FASTA sequences of these peptides, namely, Dh and XH, indicate that the aspartyl side chain is excluded from pI calculations in the case of the β-peptide. In turn, the predictive accuracy greatly improves with the chemical structure-based approach when the difference between pI of α- and β-dipeptides becomes as small as that from the experiments. Such large errors in the predicted pI for very close analogues reflect negatively on isoelectric point calculation methods as differences in pI are largely used to select the pH of mobile phases for efficient chromatographic separation of peptide analogues.^24^ In fact, if a large pI difference is wrongly predicted, the purification scientist may be misled to believe that the peptides can be separated by varying pH. In turn, when no pI difference is erroneously predicted, the scientist may miss the chance to run a quick and efficient purification. Therefore, peptide purifications are likely to be significantly slowed down by incorrect predictions.

### Accuracy of the Predicted p*K*_a_ Is Less Important than the Misassignment of Ionizable Centers

The chemical structure-based method that uses either ACDlabs or pKaMatcher p*K*_a_ predictor improves pI predictions substantially as illustrated in Figure 5. The correlation metrics are almost identical for the two p*K*_a_ predictors, even though the simpler pKaMatcher has much less granularity in capturing substituent effects on the p*K*_a_ of an ionizable group than the more prominent ACDlabs model. The latter is illustrated by the correlation between the predicted p*K*_a_ value for a set of ionizable amino acids at AstraZeneca stock that is displayed in Figure 4. The overall correlation statistics is strongly positive, *R*^2^ = 0.94 and RMSD = 0.6. However, there is often a spread of points along the *Y*-axis of ACDlabs p*K*_a_ compared to constant values along the *X*-axis of pKaMatcher predictions. The spread is significant and reaches up to 2.5 p*K*_a_ units. The fact that both methods outperformed the sequence-based approach, where the ionization of noncanonical modifications is ignored, makes us think that the inaccuracy in p*K*_a_ predictions should be less of a concern for pI calculations than ignoring the ionizable centers completely. However, one should note that the experimental validation set is rather slim and precludes from making solid conclusions.

### Model Performs Well for Natural Peptides

We validated the model on a set of natural peptides from the IPC2_peptide^43^ data set as shown in Figure 6. *R*^2^ and RMSE are 0.94 and 0.45, respectively, and they both suggest high accuracy of predictions. The calculations using only the IPC2_peptide p*K*_a_ set that was trained and validated on this IPC2_data set performed slightly better: *R*^2^ and RMSE are 0.97 and 0.25, correspondingly. However, we think that the predictions with different p*K*_a_ sets are biased to the experimental data from which they were derived. Therefore, we think that it is more instructive to utilize consensus prediction, mean pI values, and the corresponding uncertainty rather than relying on predictions with a specific p*K*_a_ set.

### Limitations of the Described Method

The linear combination of the Henderson–Hasselbalch equations is an approximation. It implies that the ionization state of one group is independent of the ionization states of other groups of the same molecule. It is a valid approximation for remote sites, where electrostatic, van der Waals, and other nonbonded interactions between the groups and electronic induction (I-), mesomeric (M-), and steric hindrance effects can be neglected. For the neighboring ionization centers, the situation becomes more complicated. Theoretically, one should calculate populations of ionization microstates (tautomers of a molecular form with a defined net charge) for each macrostate (a molecular form with a defined net charge). This becomes an exponentially difficult problem for large molecules such as peptides and proteins, and therefore, most of the p*K*_a_ prediction software limits the size of the molecule they can provide predictions for.^51,80^ The reader is pointed to a review by Fraczkiewicz on this topic.^81^ Moreover, p*K*_a_ of an ionizable group is directly affected by its molecular environment in three-dimensional (3D) space, for example, nonpolar low dielectric regions favor their neutral forms. It implies that conformational flexibility, conformational ensemble, and peptide fold affect p*K*_a_ of ionizable groups. All the mentioned effects are only indirectly reflected in the current model. The predefined sets of p*K*_a_ values, trained to reproduce experimental data, average out all of the cases in the training sets, thus, implicitly including the described effects into the p*K*_a_ values of the side chains and termini. Some p*K*_a_ sets (e.g., Gauci^60^ and ProMost^48^) provide additional granularity introducing dependency of the ionization of N- and C-termini on the side chain. Additionally, there are attempts to capture 3D conformation effect within the proposed p*K*_a_ set. For example, Toseland et al.^57^ and Grimsley et al.^55^ derived p*K*_a_ from experimental data of folded proteins, while Thurlkill et al.^56^ measured p*K*_a_ values of pentapeptides as a model of disordered protein chains. Moreover, there are physics-based models such as PropKa^82^ that are widely used to assign protonation states of 3D models of proteins and peptides. However, they require 3D conformation as an input and are rather computationally demanding and therefore fall out of the scope of this work. Overall, the approach described in this work, despite its limitations, provides reasonable accuracy for the compounds in the validation set, and thus, it is thought to be useful for peptide chemical optimization and the design of peptide purification experiments.

## Summary and Conclusions

In this work, we have described a model for the calculation of the isoelectric point of modified peptides, which we refer to as pIChemiSt. The model accepts a chemical structure as an input and calculates charge versus pH curves, isoelectric point, net charge at pH 7.4, and isoelectric interval, which is the pH interval where the charge of the peptide is negligible. The latter is useful to characterize molecules with poorly defined or uncertain isoelectric points. The model identifies noncanonical amino acids and other fragments, automatically detects ionizable groups, and predicts their p*K*_a_. The ACDlabs GALAS model is used by default for the prediction of p*K*_a_ if the user has access to the software. Alternatively, our pKaMatcher tool can be used for such a purpose, which provides accurate p*K*_a_ prediction for a set of nCAAs from the AstraZeneca reagent inventory.

We curated a data set of experimental pI data for modified peptides. For many compounds, their calculated pI values were labeled as experimental data in the Reaxys database. We scrutinized the original publication and identified that only 29 data points out of 99 were true measurements. The proposed chemical structure-based isoelectric point model was validated on the curated set of experimental data. We have shown that the chemical structure-based model significantly improves the accuracy of predictions compared to the plain sequence-based model, with R^2^ being increased from 0.74 to 0.96 and 0.95 and RMSE decreased from 1.09 to 0.44 and 0.51 where the ACDlabs and pKaMatcher tools were used for p*K*_a_ predictions of noncanonical amino acids, correspondingly. It is worth mentioning that the model correctly predicts differences between close analogues, for example, peptides with a primary amide or free acid at the C-terminus, where the sequence-based model fails.

We think that pIChemiSt can facilitate design and handling of peptide with noncanonical aminoacids, particularly, during the medicinal chemistry optimization and peptide purification.^53^

## Supporting Information Available

Defined SMARTS patterns and the raw data of the validation set compounds are listed in the SI (PDF)

## Notes

pIChemiSt is available under Apache2 license at https://github.com/AstraZeneca/peptide-tools. We used v1.2.0 of “peptide-tools” distribution, RDKit v2021.03.1, Matplotlib v3.0.3, BioPython v1.79 in this work. Dimorphite_DL v1.2.4 was accessed at https://github.com/UnixJunkie/dimorphite_dl. The code of pIR and pICalculax were accessed at https://github.com/bigbio/pIR and https://github.com/EBjerrum/pICalculax on 10/10/2022. We used version 14.50.122369 of the PhysChem module of Percepta Batch of ACDlabs software released in 2021. The IPC2_peptide data set was downloaded from http://ipc2.mimuw.edu.pl/datasets.html on 21/10/2022.

## Abbreviations

nCAA: noncanonical amino acids
pI: isoelectric point
PPI: protein–protein interactions
PT: peptide therapeutics
IEC: ion-exchange chromatography
FDA: Food and Drug Administration
PTM: post-translational modifications
HPLC: high-performance liquid chromatography
SI: supporting information;
