# Supplementary material for: pIChemiSt — Free Tool for the Calculation of Isoelectric Points of Modified Peptides
Source: J Chem Inf Model. 2022 Dec 27;63(1):187–96. doi: 10.1021/acs.jcim.2c01261 (PMC9832473; doi:10.1021/acs.jcim.2c01261)
Supplement: Supplementary file 1 — ci2c01261_si_001.pdf [file ci2c01261_si_001.pdf]

Supporting Information
pIChemiSt – Free Tool for the Calculation of Isoelectric Points of Modified Peptides

Andrey I. Frolov,[1*] Sunay V. Chankeshwara,[1] Zeyed Abdulkarim,[2] Gian Marco Ghiandoni[3]

1. Medicinal Chemistry, Research and Early Development, Cardiovascular, Renal and Metabolism (CVRM), BioPharmaceuticals R&D, AstraZeneca, Gothenburg, Sweden

2. Early Chemical Development, Pharmaceutical Sciences, BioPharmaceuticals R&D, AstraZeneca, Gothenburg, Sweden

3. Augmented DMTA Engineering, R&D IT, AstraZeneca, Cambridge, CB2 8DU, United Kingdom

* Corresponding author: andrey.frolov@astrazeneca.com

Table S1. List of SMARTS patterns implemented in pKaMatcher.

Name	SMARTS	Ionization site 1				Ionization site 2			
		atom _idx	pKa	pKa_ std	dissociation _type	atom _idx	pKa	pKa_ std	dissociation _type
12-diOH-Phenol	[OH1][c]1[c](-[O])[c][c][c][c]1	1	9.3	0.4	acid	4	13	0.4	acid
13-diOH-Phenol	[OH1][c]1[c][c](-[O])[c][c][c]1	1	8.9	0.4	acid	5	11.3	0.5	acid
14-diOH-Phenol	[OH1][c]1[c][c][c](-[O])[c][c]1	1	9.6	0.4	acid	6	11.5	0.5	acid
2-NO2-Phenol	[OH1][c]1[c](-[N](=[O])[O])[c][c][c][c]1	1	7.2	0.4	acid				
2-Hal-Phenol	[OH1:1][c:2]1[$(c-[F,Cl,Br,I]):3][c:4][c:5][c:6][cH1,!$(c-[F,Cl,Br,I]):7]1	1	8.3	0.4	acid				
2-5-Hal-Phenol	[OH1:1][c:2]1[$(c-[F,Cl,Br,I]):3][c:4][c:5][c:6][$(c-[F,Cl,Br,I]):7]1	1	6.6	0.4	acid				
Phenol	[OH1:1][c:2]1[c:3][c:4][c:5][c:6][c:7]1	1	9.7	0.4	acid				
2F-Imidazole	[nH1,$(n-[#6])]1[c](-[F])[nH0][c][cH1,$(c-[#6])]1	5	3.6	0.4	base	1	10.9	0.4	acid
2F-Imidazole	[nH1,$(n-[#6])]1[c](-[F])[nH0][cH1,$(c-[#6])][c]1	5	3.6	0.4	base	1	10.9	0.4	acid
Imidazole	[nH1,$(n-[#6])]1[c][nH0][c][cH1,$(c-[#6])]1	4	6.8	0.6	base				
Imidazole	[nH1,$(n-[#6])]1[c][nH0][cH1,$(c-[#6])][c]1	4	6.8	0.6	base				
Pyridine	[nX2:1]1[c:2][c:3][c:4][c:5][c:6]1	1	4.9	0.1	base				
Tetrazole	[nH1r5:1][nH0r5:2][nH0r5:3][nH0r5:4][cH0r5:5]	1	5.3	0.4	acid				
Tetrazole	[nH1r5:1][nH0r5:2][cH0r5:3][nH0r5:4][nH0r5:5]	1	5.3	0.4	acid				
Azide	[N+0:1]=[N+:2]=[N+0H1:3]	2	4.65	0.07	acid				
Nitro	[C,c,N,n,O,o:1]-[NX3:2](=[O:3])-[OH1:4]	3	-1000	0	acid				
NO2Guanidine	[N:1]-[C:2](=[NX2:3])-[N:4]-[N:5](=[O:6])-[O:7]	3	-0.8	0.4	base				
OGuanidine	[O:1]-[N:2]-[C:3](=[NX2:4])-[N:5]	4	7.4	0.4	base				
OGuanidine	[O:1]-[N:2]=[C:3](-[N:4])-[N:5]	4	7.4	0.4	base				
AmidineGuanidine1	[N:1]-[C:2](-[N:3])=[NX2:4]	3	12.03	1.59	base				
AmidineGuanidine2	[C:1](-[N:2])=[NX2+0:3]	2	10.04	2.13	base				

Sulfate	[SX4:1](=[O:2])(=[O:3])([O:4]-[C,c,N,n:5])-[OX2H1:6]	5	-2.36	1.3	acid				
Sulfonate	[SX4:1](=[O:2])(=[O:3])(-[C,c,N,n:4])-[OX2H1:5]	4	-1.82	1.41	acid				
Sulfinic_acid	[SX3:1](=[O:2])-[OH1:3]	2	1.79	0.44	acid				
Phenyl_carboxylic_acid	[c,n,o:1]-[C:2](=[O:3])-[OH1:4]	3	3.46	1.25	acid				
Carboxylic_acid	[CX3](=O)[OX2H1]	2	3.46	1.29	acid				
Thioic_acid	[C,c,N,n:1](=[O,S:2])-[SX2H1,OX2H1:3]	2	0.68	1.5	acid				
Phenyl_Thiol	[c,n:1]-[SX2H1:2]	1	4.98	2.61	acid				
Thiol	[C,N:1]-[SX2H1:2]	1	9.12	1.33	acid				
Phosphate	[PX4:1](=[O:2])(-[OX2H1:3])(-[O+0:4])-[OX2H1:5]	2	2.42	1.11	acid	5	6.51	0.95	acid
Internal_phosphate_polyphos_chain	[$([PX4:1](=O)([OX2][PX4](=O)([OX2])(O[H]))([OX2][PX4](=O)(O[H])([OX2])))][OH1:2]	1	0.9	1	acid				
Initial_phosphate_like_in_ATP_ADP	[$([PX4:1]([OX2][C,c,N,n])(=O)([OX2][PX4](=O)([OX2])(O[H])))][OH1]	1	2.42	1.11	acid				
Phosphonate	[PX4:1](=[O:2])(-[OX2H1:3])(-[C,c,N,n:4])-[OX2H1:5]	2	1.88	0.59	acid	5	7.25	0.85	acid
Peroxide1	[O:1]([$(C=O),$(C[Cl]),$(CF),$(C[Br]),$(CC#N):2])-[OH1:3]	2	8.74	0.76	acid				
Peroxide2	[C:1]-[O:2]-[OH1:3]	2	11.98	0.87	acid				
O=C-C=C-OH	[O:1]=[C;R:2]-[C;R:3]=[C;R:4]-[OH1:5]	4	3.55	0.8	acid				
Vinyl_alcohol	[C:1]=[C:2]-[OH1:3]	2	8.87	1.66	acid				
N-hydroxyamide	[C:1](=[O:2])-[N:3]-[OH1:4]	3	9.3	1.22	acid				
Ringed_imide1	[O,S:1]=[C;R:2]([$([#8]),$([#7]),$([#16]),$([#6][Cl]),$([#6]F),$([#6][Br]):3])-[NH1;RH1:4]([C;R:5]=[O,S:6])	3	6.45	0.56	acid				
Ringed_imide2	[O,S:1]=[C;R:2]-[NH1;RH1:3]([C;R:4]=[O,S:5])	2	8.68	1.87	acid				
Imide	[F,Cl,Br,S,s,P,p:1][#6:2][CX3:3](=[O,S:4])-[NX3H1+0:5]([CX3:6]=[O,S:7])	4	2.47	1.48	acid				
Imide2	[O,S:1]=[CX3:2]-[NX3H1+0:3]([CX3:4]=[O,S:5])	2	10.23	1.12	acid				
Amide_electronegative	[C:1](=[O:2])-[NH1:3](-[Br,Cl,I,F,S,O,N,P:4])	2	3.49	2.69	acid				
TertiaryAmide	[C:1](=[O:2])-[NH0:3]	3	-1.1	0.5	base				
Amide	[C:1](=[O:2])-[NH1:3]	3	12.01	4.51	acid				
Sulfonamide	[SX4:1](=[O:2])(=[O:3])-[NX3H1+0:4]	3	7.92	1.98	acid				
Anilines_primary	[c:1]-[NX3+0H2:2]	2	3.9	2.07	base				
Anilines_secondary	[c:1]-[NX3+0H1:2]	2	4.34	2.18	base				

Anilines_tertiary	[c:1]-[NX3+0H0:2]	2	4.17	2.01	base	
Hydroxylamine	[NX3+0;H2,H1,H0;!$(NC=[!#6]);!$(NC#[!#6]);!$(N=[!#6]):1][#8:2]	1	4.4	0.4	base	
Amines_aliph_epoor	[NX3+0;$(NCC=[!#6]);!$(NC=[!#6]);!$(NC#[!#6]):1]-[#6:2]	1	7.9	0.3	base	
Amines_primary	[NX3+0;H2;!$(NC=[!#6]);!$(NC#[!#6]):1][#6:2]	1	10.4	0.4	base	
Amines_secondary	[NX3+0;H1;!$(NC=[!#6]);!$(NC#[!#6]):1][#6:2]	1	10.8	0.4	base	
Amines_tertiary	[NX3+0;H0;!$(NC=[!#6]);!$(NC#[!#6]):1][#6:2]	1	10	0.4	base	
Phosphinic_acid	[PX4:1](=[O:2])(-[C,c,N,n,F,Cl,Br,I:3])(-[C,c,N,n,F,Cl,Br,I:4])-[OX2H1:5]	4	2.97	0.69	acid	
Phosphate_diester	[PX4:1](=[O:2])(-[OX2:3]-[C,c,N,n,F,Cl,Br,I:4])(-[O+0:5]-[C,c,N,n,F,Cl,Br,I:4])-[OX2H1:6]	6	2.73	2.54	acid	
Phosphonate_ester	[PX4:1](=[O:2])(-[OX2:3]-[C,c,N,n,F,Cl,Br,I:4])(-[C,c,N,n,F,Cl,Br,I:5])-[OX2H1:6]	5	2.09	0.45	acid	

Table S2. Calculated and experimental data of modified peptides in the validation set. The calculated pI values are reported as an average between different pKa sets for canonical amino acids.

Reaxys Nr.[a]	FASTA sequence generated from the structure[b,c]	pI exp	pIChemiSt pI with ACDlabs	pIChemiSt pI interval with ACDlabs	pIChemiSt pI with pKaMatcher	pIChemiSt pI interval with pKaMatcher	pI_fasta pI from sequence (canonical AAs)	Ref	SMILES	Molecular structure[d]
3042295	YGGFM	5.26	5.91	3.8-7.5	5.91	3.8-7.5	5.70	Bohner Lang et al.[2]	[H][C@](N)(CC1=CC=C(O)C=C1)C(=O)NCC(=O)NCC(=O)N[C@@]([H])(CC1=CC=CC=C1)C(=O)N[C@@]([H])(CCSC)C(O)=O	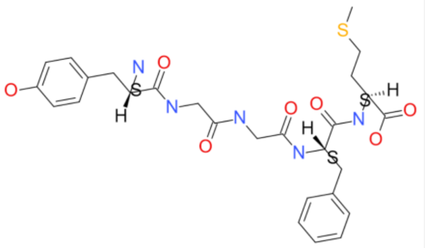
4075243	YaGFM	5.26	5.91	3.8-7.5	5.91	3.8-7.5	5.70	Bohner Lang et al.[2]	[H][C@](N)(CC1=CC=C(O)C=C1)C(=O)N[C@]([H])(C)C(=O)NCC(=O)N[C@@]([H])(CC1=CC=CC=C1)C(=O)N[C@@]([H])(CCSC)C(O)=O	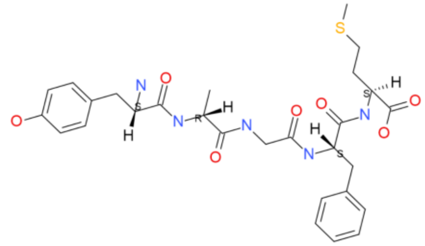

[a] The Reaxys database IDs are provided for the reference. The actual experimental data is derived from the original publications.

[b] The single letter FASTA sequences are not able to decode noncanonical modifications of the peptides in a unique fashion. In such cases the non-canonical amino acids are denoted as X or their closest canonical analogue is represented in the sequence.

[c] FASTA sequences were acquired from Bjerrum et al.[1] where they were generated from the structures using Proteax software. The sequence of compound 92374 was converted from DH to Dh to reflect the D-amino acid in the structure. In addition, we converted L- to D-alanine in the sequences of compounds 4075243, 2407420, and 3026781. Compound 40459 was not present in the set of Bjerrum et al. publication[1] and its sequence was generated manually.

[d] The molecular structure drawing were automatically generated from SMILES by RDKit software.

Reaxys Nr.[a]	FASTA sequence generated from the structure[b,c]	pI exp	pIChemiSt pI with ACDlabs	pIChemiSt pI interval with ACDlabs	pIChemiSt pI with pKaMatcher	pIChemiSt pI interval with pKaMatcher	pI_fasta pI from sequence (canonical AAs)	Ref	SMILES	Molecular structure[d]
2407420	YaGFM	8.64	9.02	8.6-9.4	9.02	8.6-9.4	5.70	Bohner Lang et al.[2]	[H][C@](N)(CC1=CC=C(O)C=C1)C(=O)N[C@]([H])(C)C(=O)NCC(=O)N[C@@]([H])(CC1=CC=CC=C1)C(=O)N(C)[C@@]([H])(CCSC)C(N)=O	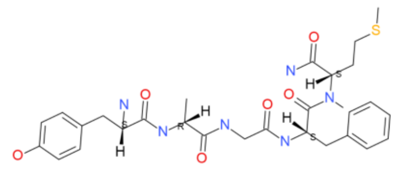
3026781	YaGFM	8.66	9.02	8.6-9.4	9.02	8.6-9.4	5.70	Bohner Lang et al.[2]	[H][C@](N)(CC1=CC=C(O)C=C1)C(=O)N[C@]([H])(C)C(=O)NCC(=O)N[C@@]([H])(CC1=CC=CC=C1)C(=O)N[C@@]([H])(CCSC)C(N)=O	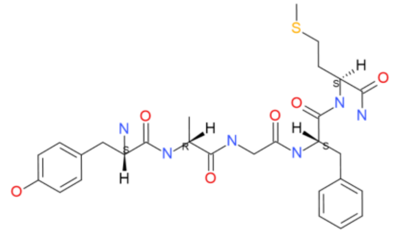
92374	Dh	4.92	5.10	4.5-5.7	5.10	4.5-5.7	4.99	Greenstein & Klemperer[3]	[H][C@](N)(CC(O)=O)C(=O)N[C@]([H])(CC1=CNC=N1)C(O)=O	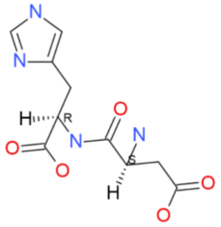
40459	XH	4.94	4.73	3.8-5.7	5.00	4.3-5.7	7.19	Greenstein & Klemperer[3]	[H][C@](N)(CC(=O)N[C@@]([H])(CC1=CNC=N1)C(O)=O)C(O)=O	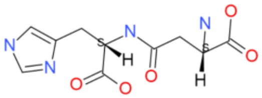
1718208	CGGCGG	4.70	4.84	4.1-5.5	5.47	4.1-6.9	5.28	Greenstein et al.[4]	[H][C@](N)(CSSC[C@]([H])(N)C(=O)NCC(=O)NCC(O)=O)C(=O)NCC(=O)NCC(O)=O	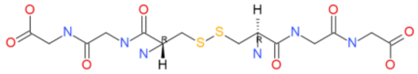

Reaxys Nr.[a]	FASTA sequence generated from the structure[b,c]	pI exp	pIChemiSt pI with ACDlabs	pIChemiSt pI interval with ACDlabs	pIChemiSt pI with pKaMatcher	pIChemiSt pI interval with pKaMatcher	pI_fasta pI from sequence (canonical AAs)	Ref	SMILES	Molecular structure[d]
1730057	CGCG	4.80	4.84	4.1-5.5	5.47	4.1-6.9	5.28	Greenstein et al. [4]	[H][C@](N)(CSSC[C@]([H])(N)C(=O)NCC(O)=O)C(=O)NCC(O)=O	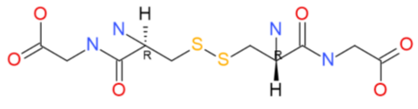
1728094	CC	4.87	5.00	2.8-7.3	5.75	4.5-6.9	5.23	Greenstein et al. [4]	[H][C@](N)(CSSC[C@]([H])(N)C(O)=O)C(O)=O	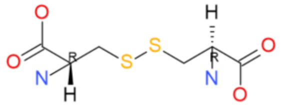
1730148	GCGC	5.30	5.94	4.2-7.3	6.00	4.5-7.3	5.27	Greenstein et al. [4]	[H][C@@](CSSC[C@]([H])(NC(=O)CN)C(O)=O)(NC(=O)CN)C(O)=O	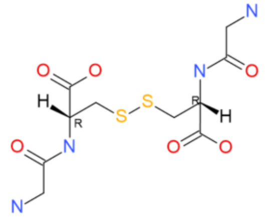
635564	EHWSYGLRPG	6.90	8.06	6.9-9.2	8.06	6.9-9.2	7.26	Heit et al.[5]	[H][C@@](CO)(NC(=O)[C@]([H])(CC1=CNC2=C1C=CC=C2)NC(=O)[C@]([H])(CC1=CNC=N1)NC(=O)[C@]1([H])CCC(=O)N1)C(=O)N[C@@]([H])(CC1=CC=C(O)C=C1)C(=O)NCC(=O)N[C@@]([H])(CC(C)C)C(=O)N[C@@]([H])(CCCNC(N)=N)C(=O)N1CCC[C@@]1([H])C(=O)NCC(O)=O	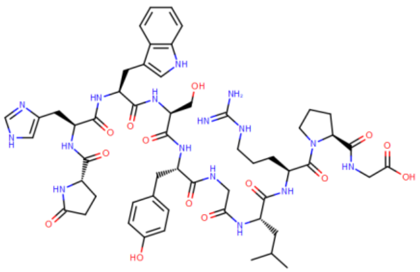

Reaxys Nr.[a]	FASTA sequence generated from the structure[b,c]	pI exp	pIChemiSt pI with ACDlabs	pIChemiSt pI interval with ACDlabs	pIChemiSt pI with pKaMatcher	pIChemiSt pI interval with pKaMatcher	pI_fasta pI from sequence (canonical AAs)	Ref	SMILES	Molecular structure[d]
741807	EHWSYGLRPG	9.60	10.98	10.4-11.5	10.98	10.4-11.5	7.26	Heit et al.[5]	[H][C@@](CO)(NC(=O)[C@]([H])(CC1=CNC2=C1C=CC=C2)NC(=O)[C@]([H])(CC1=CNC=N1)NC(=O)[C@]1([H])CCC(=O)N1)C(=O)N[C@@]([H])(CC1=CC=C(O)C=C1)C(=O)NCC(=O)N[C@@]([H])(CC(C)C)C(=O)N[C@@]([H])(CCCNC(N)=N)C(=O)N1CCC[C@@]1([H])C(=O)NCC(N)=O	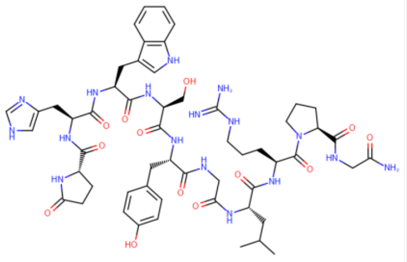
9185435	GCHHHHHHHHH	7.74	7.86	7.6-8.1	7.86	7.6-8.1	7.53	Shimura et al.[6]	CN(C)C1=CC=C2C(OC3=C\C(\C=CC3=C2C2=C(C=C(NC(=O)CSC[C@H](NC(=O)CN)C(=O)N[C@@H](CC3=CNC=N3)C(=O)N[C@@H](CC3=CNC=N3)C(=O)N[C@@H](CC3=CNC=N3)C(=O)N[C@@H](CC3=CNC=N3)C(=O)N[C@@H](CC3=CNC=N3)C(=O)N[C@@H](CC3=CNC=N3)C(=O)N[C@@H](CC3=CNC=N3)C(=O)N[C@@H](CC3=CNC=N3)C(=O)N[C@@H](CC3=CNC=N3)C(O)=O)C=C2)C([O-])=O)=[N+](/C)C)=C1	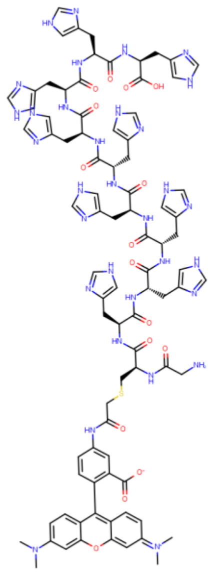

Reaxys Nr.[a]	FASTA sequence generated from the structure[b,c]	pI exp	pIChemiSt pI with ACDlabs	pIChemiSt pI interval with ACDlabs	pIChemiSt pI with pKaMatcher	pIChemiSt pI interval with pKaMatcher	pI_fasta pI from sequence (canonical AAs)	Ref	SMILES	Molecular structure[d]
9185305	GCHEHEHEHE	5.53	5.34	5.3-5.4	5.36	5.3-5.4	5.30	Shimura et al.[6]	CN(C)C1=CC=C2C(OC3=C\C(\C=CC3=C2C2=C(C=C(NC(=O)CSC[C@H](NC(=O)CN)C(=O)NC(CC3=CNC=N3)C(=O)N[C@@H](CCC(O)=O)C(=O)N[C@@H](CC3=CNC=N3)C(=O)N[C@@H](CCC(O)=O)C(=O)N[C@@H](CC3=CNC=N3)C(=O)N[C@@H](CCC(O)=O)C(=O)N[C@@H](CC3=CNC=N3)C(=O)N[C@@H](CCC(O)=O)C(O)=O)C=C2)C([O-])=O)=[N+](/C)C)=C1	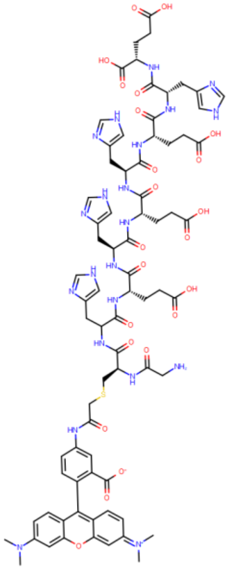
9185248	KCEYYKKY	8.77	8.83	8.8-9.0	8.81	8.7-8.9	8.83	Shimura et al.[6]	CN(C)C1=CC=C2C(OC3=C\C(\C=CC3=C2C2=C(C=C(NC(=O)CSC[C@H](NC(=O)[C@@H](N)CCCCNC(=O)CN)C(=O)N[C@@H](CCC(O)=O)C(=O)N[C@@H](CC3=CC=C(O)C=C3)C(=O)N[C@@H](CC3=CC=C(O)C=C3)C(=O)N[C@@H](CCCCN)C(=O)N[C@@H](CCCCN)C(=O)N[C@@H](CC3=CC=C(O)C=C3)C(O)=O)C=C2)C([O-	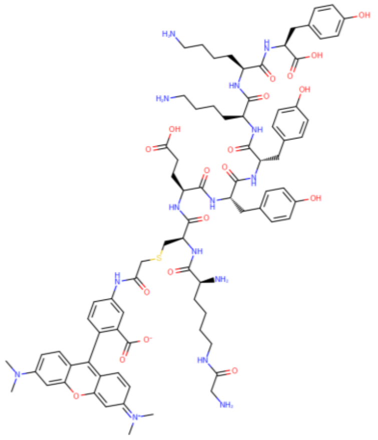

Reaxys Nr.[a]	FASTA sequence generated from the structure[b,c]	pI exp	pIChemiSt pI with ACDlabs	pIChemiSt pI interval with ACDlabs	pIChemiSt pI with pKaMatcher	pIChemiSt pI interval with pKaMatcher	pI_fasta pI from sequence (canonical AAs)	Ref	SMILES	Molecular structure[d]
									])=O)=[N+](/C)C)=C1	
9185114	GCHEHEHEH	6.18	5.85	5.9-6.0	5.86	5.8-5.9	5.78	Shimura et al.[6]	CN(C)C1=CC=C2C(OC3=C\C(\C=CC3=C2C2=C(C=C(NC(=O)CSC[C@H](NC(=O)CN)C(=O)NC(CC3=CNC=N3)C(=O)N[C@@H](CCC(O)=O)C(=O)N[C@@H](CC3=CNC=N3)C(=O)N[C@@H](CCC(O)=O)C(=O)N[C@@H](CC3=CNC=N3)C(=O)N[C@@H](CCC(O)=O)C(=O)N[C@@H](CC3=CNC=N3)C(O)=O)C=C2)C([O-])=O)=[N+](/C)C)=C1	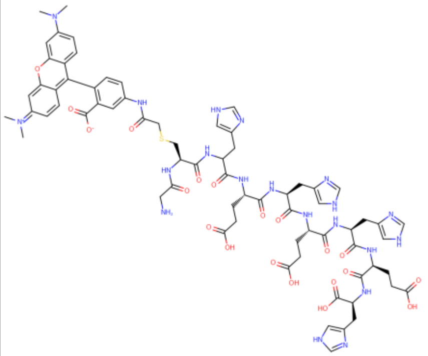
9184758	GCYYYKK	9.56	9.38	9.4-9.5	9.38	9.4-9.5	8.90	Shimura et al.[6]	CN(C)C1=CC=C2C(OC3=C\C(\C=CC3=C2C2=C(C=C(NC(=O)CSC[C@H](NC(=O)CN)C(=O)N[C@@H](CC3=CC=C(O)C=C3)C(=O)N[C@@H](CC3=CC=C(O)C=C3)C(=O)N[C@@H](CC3=CC=C(O)C=C3)C(=O)N[C@@H](CCCCN)C(=O)N[C@@H](CCCCN)C(O)=O)C=C2)C([O-])=O)=[N+](/C)C)=C1	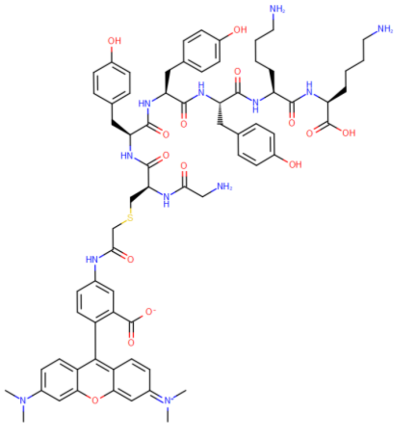

Reaxys Nr.[a]	FASTA sequence generated from the structure[b,c]	pI exp	pIChemiSt pI with ACDlabs	pIChemiSt pI interval with ACDlabs	pIChemiSt pI with pKaMatcher	pIChemiSt pI interval with pKaMatcher	pI_fasta pI from sequence (canonical AAs)	Ref	SMILES	Molecular structure[d]
9184651	GCEHHHR	7.58	7.55	7.2-7.9	7.56	7.2-7.9	7.22	Shimura et al.[6]	CN(C)C1=CC=C2C(OC3=C\C(\C=CC3=C2C2=C(C=C(NC(=O)CSC[C@H](NC(=O)CN)C(=O)N[C@@H](CCC(O)=O)C(=O)N[C@@H](CC3=CNC=N3)C(=O)N[C@@H](CC3=CNC=N3)C(=O)N[C@@H](CC3=CNC=N3)C(=O)N[C@@H](CCCNC(N)=N)C(O)=O)C=C2)C([O-])=O)=[N+](/C)C)=C1	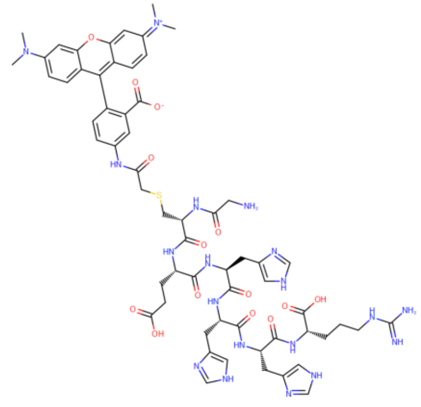
9184432	KCKKE	8.21	8.55	8.4-8.6	8.51	8.5-8.7	9.14	Shimura et al.[6]	CN(C)C1=CC=C2C(OC3=C\C(\C=CC3=C2C2=C(C=C(NC(=O)CSC[C@H](NC(=O)[C@@H](N)CCCCNC(=O)CN)C(=O)N[C@@H](CCCCN)C(=O)N[C@@H](CCCCNC(=O)CN)C(=O)N[C@@H](CCC(O)=O)C(O)=O)C=C2)C([O-])=O)=[N+](/C)C)=C1	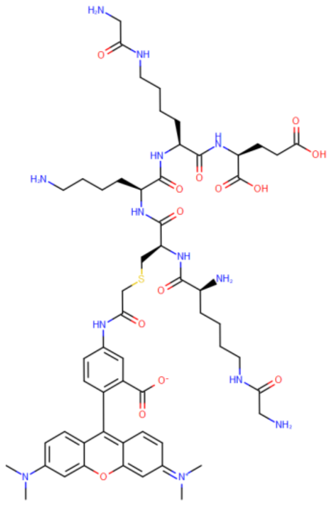

Reaxys Nr.[a]	FASTA sequence generated from the structure[b,c]	pI exp	pIChemiSt pI with ACDlabs	pIChemiSt pI interval with ACDlabs	pIChemiSt pI with pKaMatcher	pIChemiSt pI interval with pKaMatcher	pI_fasta pI from sequence (canonical AAs)	Ref	SMILES	Molecular structure[d]
9183738	GCEHR	7.38	7.30	6.8-7.8	7.30	6.8-7.8	6.93	Shimura et al.[6]	CN(C)C1=CC=C2C(OC3=C\C(\C=CC3=C2C2=C(C=C(NC(=O)CSC[C@H](NC(=O)CN)C(=O)N[C@@H](CCC(O)=O)C(=O)N[C@@H](CC3=CNC=N3)C(=O)N[C@@H](CCCNC(N)=N)C(O)=O)C=C2)C([O-])=O)=[N+](/C)C)=C1	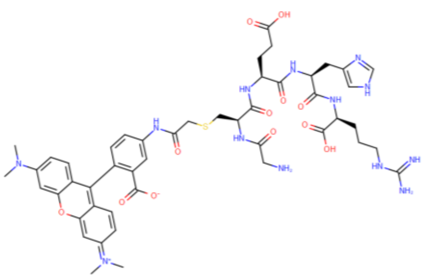
9183925	GCYKR	10.12	9.99	9.9-10.1	9.99	9.9-10.1	9.20	Shimura et al.[6]	CN(C)C1=CC=C2C(OC3=C\C(\C=CC3=C2C2=C(C=C(NC(=O)CSC[C@H](NC(=O)CN)C(=O)N[C@@H](CC3=CC=C(O)C=C3)C(=O)N[C@@H](CCCCN)C(=O)N[C@@H](CCCNC(N)=N)C(O)=O)C=C2)C([O-])=O)=[N+](/C)C)=C1	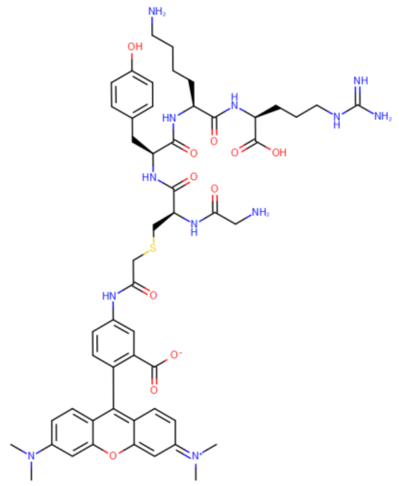

Reaxys Nr.[a]	FASTA sequence generated from the structure[b,c]	pI exp	pIChemiSt pI with ACDlabs	pIChemiSt pI interval with ACDlabs	pIChemiSt pI with pKaMatcher	pIChemiSt pI interval with pKaMatcher	pI_fasta pI from sequence (canonical AAs)	Ref	SMILES	Molecular structure[d]
9183675	GCYKK	9.94	9.74	9.7-9.8	9.74	9.7-9.8	9.08	Shimura et al.[6]	CN(C)C1=CC=C2C(OC3=C\C(\C=CC3=C2C2=C(C=C(NC(=O)CSC[C@H](NC(=O)CN)C(=O)N[C@@H](CC3=CC=C(O)C=C3)C(=O)N[C@@H](CCCCN)C(=O)N[C@@H](CCCCN)C(O)=O)C=C2)C([O-])=O)=[N+](/C)C)=C1	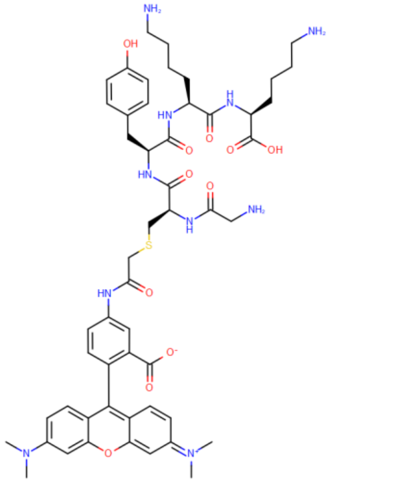
9183604	GCDDR	4.23	4.29	4.2-4.3	4.39	4.3-4.5	3.97	Shimura et al.[6]	CN(C)C1=CC=C2C(OC3=C\C(\C=CC3=C2C2=C(C=C(NC(=O)CSC[C@H](NC(=O)CN)C(=O)N[C@@H](CC(O)=O)C(=O)N[C@@H](CC(O)=O)C(=O)N[C@@H](CCCNC(N)=N)C(O)=O)C=C2)C([O-])=O)=[N+](/C)C)=C1	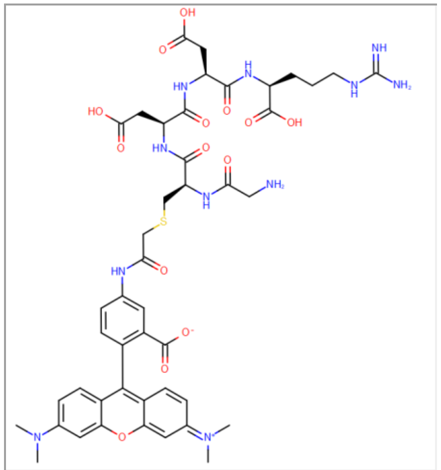

Reaxys Nr.[a]	FASTA sequence generated from the structure[b,c]	pI exp	pIChemiSt pI with ACDlabs	pIChemiSt pI interval with ACDlabs	pIChemiSt pI with pKaMatcher	pIChemiSt pI interval with pKaMatcher	pI_fasta pI from sequence (canonical AAs)	Ref	SMILES	Molecular structure[d]
9185455	GCEHEHEHEKE	4.99	4.95	4.9-5.0	4.98	4.9-5.0	4.89	Shimura et al.[6]	CN(C)C1=CC=C2C(OC3=C\C(\C=CC3=C2C2=C(C=C(NC(=O)CSC[C@H](NC(=O)CN)C(=O)N[C@@H](CCC(O)=O)C(=O)N[C@@H](CC3=CNC=N3)C(=O)N[C@@H](CCC(O)=O)C(=O)N[C@@H](CC3=CNC=N3)C(=O)N[C@@H](CCC(O)=O)C(=O)N[C@@H](CC3=CNC=N3)C(=O)N[C@@H](CCC(O)=O)C(=O)N[C@@H](CCCCN)C(=O)N[C@@H](CCC(O)=O)C(O)=O)C=C2)C([O-])=O)=[N+](/C)C)=C1	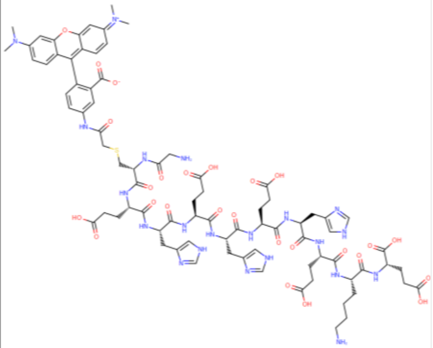

Reaxys Nr.[a]	FASTA sequence generated from the structure[b,c]	pI exp	pIChemiSt pI with ACDlabs	pIChemiSt pI interval with ACDlabs	pIChemiSt pI with pKaMatcher	pIChemiSt pI interval with pKaMatcher	pI_fasta pI from sequence (canonical AAs)	Ref	SMILES	Molecular structure[d]
9183506	GCEEH	4.50	4.58	4.5-4.6	4.64	4.6-4.7	4.36	Shimura et al.[6]	CN(C)C1=CC=C2C(OC3=C\C(\C=CC3=C2C2=C(C=C(NC(=O)CSC[C@H](NC(=O)CN)C(=O)N[C@@H](CCC(O)=O)C(=O)N[C@@H](CCC(O)=O)C(=O)N[C@@H](CC3=CNC=N3)C(O)=O)C=C2)C([O-])=O)=[N+](/C)C)=C1	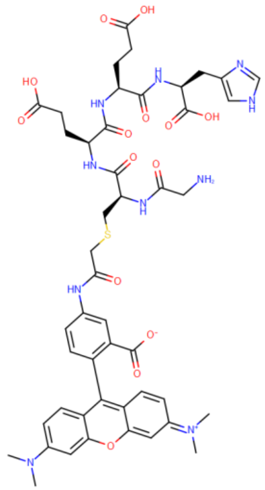
9183232	GCDDD	3.64	3.67	3.5-3.6	3.76	3.7-3.8	3.12	Shimura et al.[6]	CN(C)C1=CC=C2C(OC3=C\C(\C=CC3=C2C2=C(C=C(NC(=O)CSC[C@H](NC(=O)CN)C(=O)N[C@@H](CC(O)=O)C(=O)N[C@@H](CC(O)=O)C(=O)N[C@@H](CC(O)=O)C(O)=O)C=C2)C([O-])=O)=[N+](/C)C)=C1	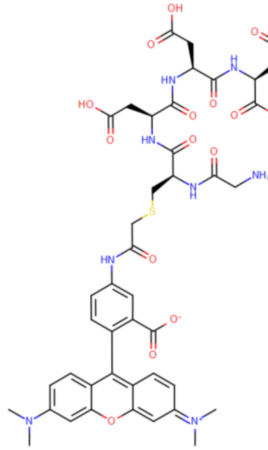

Reaxys Nr.[a]	FASTA sequence generated from the structure[b,c]	pI exp	pIChemiSt pI with ACDlabs	pIChemiSt pI interval with ACDlabs	pIChemiSt pI with pKaMatcher	pIChemiSt pI interval with pKaMatcher	pI_fasta pI from sequence (canonical AAs)	Ref	SMILES	Molecular structure[d]
9180212	GCEE	3.99	4.10	4.1-4.2	4.20	4.1-4.2	3.58	Shimura et al.[6]	CN(C)c(cc1)cc(c12)oc3c(ccc(c3)=[N+](C)C)c2-c4c(C([O-])=O)cc(cc4)NC(=O)CSC[C@H](NC(=O)CN)C(=O)N[C@H](CCC(=O)O)C(=O)N[C@@H](C(=O)O)CCC(=O)O	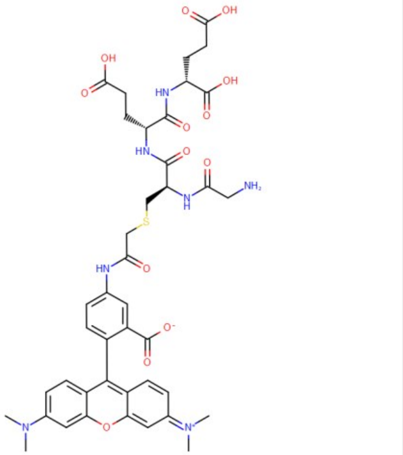
9184333	GCYYKK	9.70	9.52	9.5-9.6	9.52	9.5-9.6	8.98	Shimura et al.[6]	CN(C)C1=CC=C2C(OC3=C\C(\C=CC3=C2C2=C(C=C(NC(=O)CSC[C@H](NC(=O)CN)C(=O)N[C@@H](CC3=CC=C(O)C=C3)C(=O)N[C@@H](CC3=CC=C(O)C=C3)C(=O)N[C@@H](CCCCN)C(=O)N[C@@H](CCCCN)C(O)=O)C=C2)C([O-])=O)=[N+](/C)C)=C1	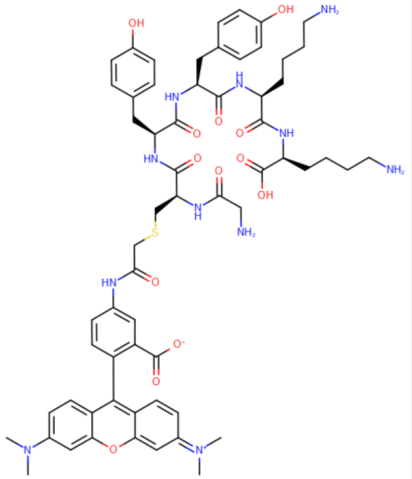

Reaxys Nr.[a]	FASTA sequence generated from the structure[b,c]	pI exp	pIChemiSt pI with ACDlabs	pIChemiSt pI interval with ACDlabs	pIChemiSt pI with pKaMatcher	pIChemiSt pI interval with pKaMatcher	pI_fasta pI from sequence (canonical AAs)	Ref	SMILES	Molecular structure[d]
9183516	GCEHH	6.86	6.29	6.2-6.4	6.29	6.2-6.4	6.13	Shimura et al.[6]	CN(C)C1=CC=C2C(OC3=C\C(\C=CC3=C2C2=C(C=C(NC(=O)CSC[C@H](NC(=O)CN)C(=O)N[C@@H](CCC(O)=O)C(=O)N[C@@H](CC3=CNC=N3)C(=O)N[C@@H](CC3=CNC=N3)C(O)=O)C=C2)C([O-])=O)=[N+](/C)C)=C1	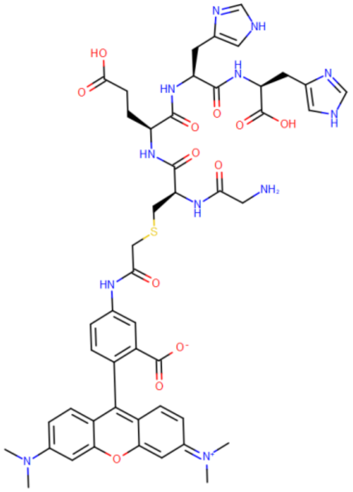

References

(1) Bjerrum, E. J.; Jensen, J. H.; Tolborg, J. L. pICalculax: Improved Prediction of Isoelectric Point for Modified Peptides. *J. Chem. Inf. Model.* **2017**, *57*, 1723–1727. https://doi.org/10.1021/acs.jcim.7b00030.

(2) Bohner Lang, V.; Langguth, P.; Ottiger, C.; Wunderli-Allenspach, H.; Rognan, D.; Rothen-Rutishauser, B.; Perriard, J.-C.; Lang, S.; Biber, J.; Merkle, H. P. Structure–Permeation Relations of Met-Enkephalin Peptide Analogues on Absorption and Secretion Mechanisms in Caco-2 Monolayers. *J. Pharm. Sci.* **1997**, *86*, 846–853. https://doi.org/10.1021/js960387x.

(3) Greenstein, J. P.; Klemperer, F. W. Aspartylhistidine. *J. Biol. Chem.* **1939**, *128*, 245–250. https://doi.org/10.1016/S0021-9258(18)73747-2.

(4) Greenstein, J. P.; Klemperer, F. W.; Wyman, J. Further Studies On The Physical Chemistry Of Cystine Peptides. *J. Biol. Chem.* **1939**, *129*, 681–692. https://doi.org/10.1016/S0021-9258(18)73632-6.

(5) Heit, M. C.; McFarland, A.; Bock, R.; Riviere, J. E. Isoelectric Focusing and Capillary Zone Electrophoretic Studies Using Luteinizing Hormone Releasing Hormone and Its Analog. *J. Pharm. Sci.* **1994**, *83*, 654–656. https://doi.org/10.1002/jps.2600830512.

(6) Shimura, K.; Kamiya, K.; Matsumoto, H.; Kasai, K. Fluorescence-Labeled Peptide PI Markers for Capillary Isoelectric Focusing. *Anal. Chem.* **2002**, *74*, 1046–1053. https://doi.org/10.1021/ac0108010.
